# Development and evaluation of an immunochromatographic strip for rapid detection of porcine hemagglutinating encephalomyelitis virus

**DOI:** 10.1186/1743-422X-9-172

**Published:** 2012-08-24

**Authors:** Keyan Chen, Kui Zhao, Deguang Song, Wenqi He, Wei Gao, Chuanbo Zhao, Chengli Wang, Feng Gao

**Affiliations:** 1 College of Animal Science and Veterinary Medicine, Jilin University, Changchun, 130062, China; 2 Department of Laboratory Animal, General Hospital of Shenyang Military Area Command, Shenyang, 110840, China

**Keywords:** Hemagglutinating encephalomyelitis virus, Coronavirus, Immunochromatographic strip, Detection

## Abstract

**Background:**

The incidence of PHE among pigs in many countries is on the rise, and it has caused great economic losses to the pig industry. Therefore, the development of a sensitive, specific, and easily-performed assay is crucial for the rapid detection and surveillance of PHE-CoV infection and transmission.

**Results:**

An immunochromatographic strip was developed for the detection of PHE-CoV. The colloidal gold-labeled MAb 4D4 was used as the detection reagent, and the MAb 1E2 and goat anti-mouse IgG coated the strip's test and control lines, respectively. The immunochromatographic strip was capable of specifically detecting PHE-CoV with a HA unit of 2 within 10 min. Storage of the strips at room temperature for 6 months or at 4°C for 12 months did not change their sensitivity or specificity. Using RT-PCR as a reference test, the relative specificity and sensitivity of the immunochromatographic strip were determined to be 100% and 97.78%, respectively. There was an excellent agreement between the results obtained by RT-PCR and the immunochromatographic strips (kappa = 0.976). Additionally, there was a strong agreement between the sandwich enzyme-linked immunosorbent assay (ELISA) and immunochromatographic strips (Kappa = 0.976). When the immunochromatographic strips were used for diagnosing PHE-CoV infection in the Jilin Province, the PHE-CoV-positive rate ranged from 61.54% in the Jilin district to 17.95% in the Songyuan district.

**Conclusions:**

Based on its high specificity, sensitivity, and stability, the immunochromatographic strip would be suitable for on-site detection of PHE-CoV for surveillance and epidemiological purposes.

## Background

Porcine hemagglutinating encephalomyelitis (PHE) is an infectious disease primarily affecting pigs under 3 weeks of age, causing vomiting, exhaustion, and obvious neurological symptoms [1]. The mortality rate varies between 20-100% [2]. The disease is caused by PHE coronavirus (PHE-CoV), which comprises a single strain and is the only known neurotropic CoV affecting pigs [3]. PHE-CoV was isolated for the first time in vivo from breast-feeding pigs suffering from encephalomyelitis in Canada [4]. In 1969, an antigenically identical virus was isolated in England from suckling pigs presenting with anorexia, depression, and vomiting, but without clear signs of encephalomyelitis [5]. Surviving animals remained stunted in growth, and the condition was therefore called ‘vomiting and wasting disease’ (VWD). In China, PHE-CoV was first reported in 1986, and it was later described both on the mainland and in the Taiwan Province [1,6]. The infection has also been reported in the major pig raising countries of Europe, Asia and North America, where it seemed to be endemic with no clinical outbreaks [7-9].

Currently, the incidence of PHE among pigs in many countries is on the rise, and it has caused great economic losses to the pig industry. In 2001, PHE-CoV was isolated from newborn and early-weaned pigs with vomiting and posterior paralysis on a Canadian farm [10]. In 2007, this disease occurred twice in pig farms in the Jilin Province, with incidence rates among 20-day-old piglets as high as 100% and reported mortality rates ranging from 48% and 100% [11]. In August 2006, some pig farms in Argentina experienced outbreaks of this disease, leading to 1226 deaths, morbidity rates as high as 52.6% and a mortality rate of 16.9% [12]. Therefore, early detection and control of PHE-CoV infection would be significant both from an economic and health viewpoint.

At present, various laboratory methods are available for the detection and surveillance of PHE-CoV, including virus isolation [1], hemagglutination/hemagglutination inhibition (HA/HI) tests [13], immunohistochemistry (IHC) assays [10], and molecular tools such as nested-polymerase chain reaction (nested PCR) and reverse transcriptase-polymerase chain reaction (RT-PCR) that enable detection of specific CoV RNA sequences from infected tissues [14,15]. However, these detection methods are laborious, time-consuming, and require laboratory procedures or special equipment, making them unsuitable for on-site inspection. Current detection strategies are also insufficient to meet the needs of emergent management after PHE outbreaks, thus restricting their application to veterinary clinical diagnosis. Therefore, the development of a sensitive, specific, and easily-performed assay is crucial for the rapid detection and surveillance of PHE-CoV infection and transmission.

An immunochromatographic assay is a unique immunoassay developed in 1980 in which a cellulose membrane is used as the carrier and a colloidal gold-labeled antigen or antibody is used as the tracer [16]. It combines the immune response with chromatographic theory in a test that is simple and quick, providing specific, sensitive, and clear results with simple or no instrumentation. Thus, it is suitable for testing clinical samples on site, in clinics and in locales where medical treatment and laboratory facilities are not available. An immunochromatographic assay has been widely used for animal quarantine and medical reasons [17,18]. (1–3)In this study, an immunochromatographic strip with high sensitivity and specificity was developed for the detection of PHE-CoV, combining monoclonal antibody (MAb) and colloidal gold immunochromatography (GICA), and the resulting product is suitable for the surveillance of PHE-CoV.

## Methods

### Animals and samples

Balb/C mice were purchased from the Laboratory Animal Center of General Hospital of Shenyang Military Area Command in China. Animal immunization experiments were performed in accordance with the guidelines for animal experimentation of the General Hospital of Shenyang Military Area Command. The animals were maintained under pathogen-free conditions. The field samples (brain tissue samples from deceased piglets and nasal cavity or throat swabs from ill piglets) were provided by Jilin Center for Disease Control and Prevention in order to perform general surveillance on the PHE-CoV infection.

### Virus strain

The virus strain used in this study was PHE-CoV-67 N (GenBank accession no. AY078417). The viruses were propagated and passaged in porcine kidney epithelial (PK)-15 cells [19], and purified by sucrose density gradient centrifugation. The viruses were stored at −80°C until needed.

### Preparation of anti-PHE-CoV Mab

The procedure employed for the production of the monoclonal antibodies (MAb) against the HE and S proteins of PHE-CoV were based on the protocol of Kohler and Milstein [12],and the recombinant S protein of PHE-CoV were produced as previously described [20]. Briefly, Balb/C mice at 8 weeks of age were immunized subcutaneously with 0.2 ml (2^8^ units of HA) of PHE-CoV virus purified by sucrose density gradient centrifugation and emulsified 1:1 with Freund's complete adjuvant (Sigma, St. Louis, MO). The mice were boosted three times with the same amount of antigen in 50% Freund's incomplete adjuvant (Sigma, St. Louis, MO) every 2 weeks, followed by an intraperitoneal injection of 0.4 ml PHE-CoV. Three days later, their splenic mononuclear cells were isolated and fused with murine myeloma cells (SP2/0) using 50% polyethylene glycol (PEG)-1000 (Sigma, St. Louis, MO). The hybridomas were generated through the selection of HAT (Sigma, St. Louis, MO) medium and screened using a recombinant S protein-based Enzyme-linked immunosorbent assay (ELISA) and HI assays. The positive hybridoma cells were cloned by a limiting dilution to obtain four strains of secretory positive antibody hybridoma cells. The stable hybridoma clones of the immunoglobulin G1 MAb 4D4 and 1E2 were injected into Balb/C nude mice and the mouse abdominal dropsy was purified by sequential precipitation with caprylic acid [21] and ammonium sulfate and dialyzed against phosphate buffer (0.01 M, pH 7.4) at 4°C. Their purities were used in Western blot analysis.

### The sandwich enzyme-linked immunosorbent assay (ELISA)

The ELISA assay was based on the MAb of anti-PHE-CoV for the detection of PHE-CoV. The purified MAb 4D4 of PHE-CoV, diluted to a final concentration of 8.4 μg/ml with carbonate buffer (0.05 M, pH 9.6), was used as the capture antibody in ELISA for coating 96-well microtiter plates (Costar Corning Inc., Corning, NY.) with 100 μl per well. The plate was incubated overnight at 4°C. The plates were washed 3 times with PBS-tween20 (PBST), and nonspecific binding sites were blocked with 3% (w/v) bovine serum albumin (BSA) (Sigma, St. Louis, MO) in PBST (200 μl/well) for 2 h at 37°C. After washing the plates, the samples of the homogenate grind suspension or the supernatant of the cell cultures were diluted 1:100 with PBST containing 2% (w/v) BSA, and 100 μl was added into the coated well. The PHE-CoV and PBST standards were added as positive and negative controls, respectively, and the samples were incubated for 1.5 h at 37°C. After washing the plates, the MAb 1E2 (8 μg/ml) was added to each well at 100 μl per well, the samples were incubated for 1.5 h at 37°C, and the plates were washed 3 times with PBST. Horseradish peroxidase-conjugated goat anti-mouse IgG (Sigma, St. Louis, MO) was added into each well at a working concentration of 1:4000, incubated for 1 h at 37°C, and the plates were washed 4 times with PBST. One hundred microliters of substrate solution o-phenylenediamine (OPD containing H_2_O_2_) was added to each well, and the color reaction was developed in the dark for 10 minutes at room temperature. The reaction was then stopped with 2 M of H_2_SO_4_ 50 μl/hole, and the absorbance was read at OD_490_ with a Universal Microplate Reader (Bio-Rad Laboratories Inc., Richmond, CA).

### Preparation of colloidal gold and colloidal gold-MAb conjugate

Colloidal gold was prepared as previously reported [22], with minor modifications. Briefly, 100 ml of 0.01% (wt/vol) HAuCl_4_ in doubly distilled water in a 250-ml siliconized conical flask was heated to boiling in a microwave oven, and then 1.4 ml 1% trisodium citrate was added to the solution. After the colloidal gold solution was boiled for an additional 14 min, it turned a cardinal red color and was allowed to cool gradually and was stored at 4°C in a dark-colored glass bottle. The pH of the colloidal gold was adjusted to 8.4 with 1% potassium carbonate (wt/vol), followed by sub-installing 11 tubes with 1 ml colloidal gold solution each, to which were added 5 μg, 10 μg, 15 μg, 20 μg, 25 μg, 30 μg, 35 μg, 40 μg, or 45 μg MAb 4D4. Tubes were shaken for 20 min, and then 0.1 ml of 10% NaCl solution was added to each tube and mixed. Two hours later, results were recorded.

The MAb 4D4 antibody (37.7 μl, 9.08 mg/ml) was added dropwise into 10 ml of colloidal gold solution on a magnetic stirring apparatus for 30 min, stood at 4°C for 30 min, and then 1 ml 10% (wt/vol) BSA was added to block excess reactivity of the gold colloid. The mixture was then stirred on the magnetic stirring apparatus for an additional 30 min and stored at 4°C for 2 h. After the mixture was centrifuged at 3,000 × g at 4°C for 30 min, the supernatant was centrifuged at 14,000 × g at 4°C for 45 min, and the resulting conjugate pellet was suspended in 10 mM borax buffer (pH 8.0) containing 2% (wt/vol) BSA and 0.05% NaN_3_. The sizes and shapes of the unconjugated colloidal gold and colloidal gold conjugated to antibodies were characterized using transmission electron microscopy.

### Preparation of the immunochromatographic strip

**Figure 1:**
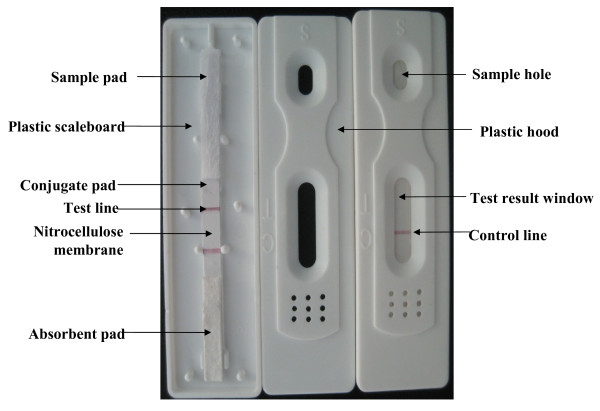
**Schematic diagram of the immunochromatographic strip.** The conjugate pad is coated with colloidal gold-labeled MAb 4D4, MAb 1E2 and goat anti-mouse IgG are immobilized at the test line and control line positions, respectively.

The immunochromatographic test device consisted of a plastic support to which an immunochromatographic strip composed of a sample pad, a conjugate pad, a nitrocellulose (NC) membrane, and an absorbent pad were mounted. The colloidal gold-labeled MAb 4D4 solution was dispensed onto glass fiber paper at a speed of 50 μl per cm using an XYZ3000 Dispense Workstation (BECKMAN, USA), and the conjugate pad was dried under vacuum. The MAb 1E2 (4.8 mg/ml) or the goat anti-mouse antibody (1 mg/ml) was dispensed at the test or the control line on the NC membrane, at a rate of 0.8 μl/cm and a speed of 4 cm/s using XYZ-3000, and the membrane was dried under vacuum and stored at 4°C. The sample pad, pretreated conjugate pad, NC membrane, and absorbent pads were glued together on a support board and assembled into a test strip plate. Then the strip plate was cut into 4-mm-wide pieces using an LN-5000 cutting machine (BECKMAN, USA). In addition, a sample pad completed the assembly with 1.0- to 1.5-mm overlap sequentially by mounting on the conjugate plastic card (Figure 1). The strips were stored in dry conditions at 4°C until required.

### Detection principle and test procedure

**Figure 2:**
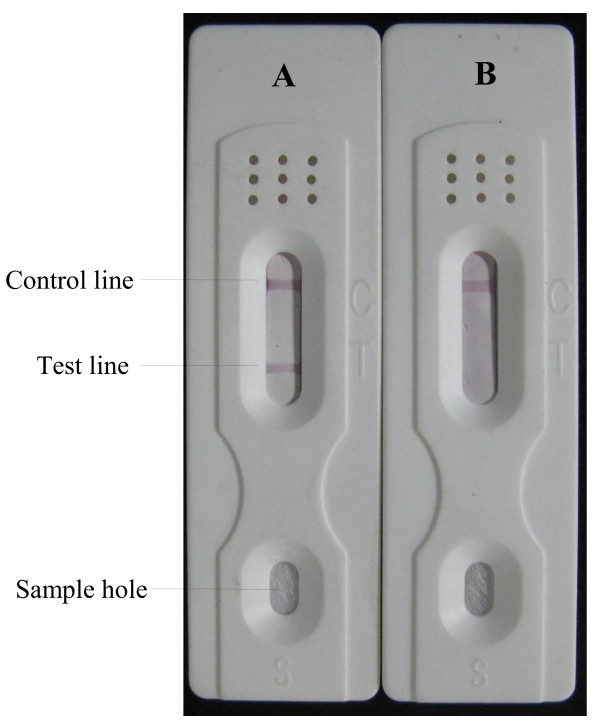
**Interpretation of the results of the immunochromatographic strip.A**, positive (two red bands in the readout zone); **B**, negative (only one red band in the control line area).

During testing, approximately 100 μl supernatant of the antigen samples were added to the sample hole of the immunochromatographic strip, and this liquid rapidly diffused into the conjugate pad. For positive samples, PHE-CoV was captured by MAb 4D4 through percolation in the nitrocellulose membrane, while in the test line, the formation of a colloidal gold MAb 4D4-PHE-CoV-MAb 1E2 complex caused the appearance of a red line; for negative samples, the test line zone did not form a colloidal gold MAb 4D4-PHE-CoV-MAb 1E2 complex, and therefore no red line was evident. As a control, both the negative and positive sample control lines turned red by forming a colloidal gold MAb 4D4-goat anti-mouse IgG complex; otherwise, the test results were invalid. The immunochromatographic strip is illustrated in Figure 2.

### Specificity and sensitivity of the immunochromatographic strip

To evaluate the specificity of the immunochromatographic strips, PHE-CoV and other viruses, including TGEV, PEDV, PRV, HCV, BCV, MHV and HCV-OC43 were simultaneously tested using the test strips. The virus samples of PHE-CoV (128 units of HA) was diluted to 1:2, 1:4, 1:8, 1:16, 1:32, 1:64, 1:128, 1:256, and 1:512 with 10 mmol/l borate buffer solution (pH 8.0), and these samples were simultaneously tested using the immunochromatographic strips to evaluate the strips’ sensitivity. The same procedure was repeated three times by different operators.

### Reproducibility and stability of the immunochromatographic strip

To determine the reproducibility of the strip, the same batch and five different batches of the immunochromatographic strip were used to detect PHE-CoV. All samples were repeated 5 times and the data were collected. The immunochromatographic strips were stored at room temperature or at 4°C and used for testing positive (32 HA units) and negative samples every 2 months, to determine the stability of the test.

### Agreement between the immunochromatographic strip and reference methods

To evaluate the correlation between the immunochromatographic strip and reference methods, a total of 178 brain tissue samples were collected from deceased piglets with suspected PHE-CoV infection from several pig farms in the Jilin Province. A 500 mg of brain tissue was measured and homogenized, then suspended 1:10 with PBS, followed by centrifuging at 7000 × g for 10 min. The supernatant was collected for testing by ELISA, the immunochromatographic strips, RT-PCR. The detection assay of PHE-CoV by RT-PCR were established as previously described [23]. The kappa statistic [24] was used to measure the strength of agreement among the results between the immunochromatographic strips, ELISA, RT-PCR. Kappa statistic values of >0.75, 0.40 to 0.75, and <0.40 represented excellent agreement, good to fair agreement, and poor agreement, respectively [25].

### Diagnosis of PHE-CoV infection in the field

The immunochromatographic strips were applied to the diagnosis of PHE-CoV infection in the field. A total of 468 nasal cavity or throat swabs were collected from approximately 1- to 3-week-old piglets, with vomiting and neurological symptoms consistent with PHE-CoV infection, from 12 herds in the Changchun, Jilin, Songyuan, Siping, Baishan, and Liaoyuan districts of the Jilin Province, China in 2010.

## Results

### Preparation of anti-PHE-CoV Mab

**Figure 3:**
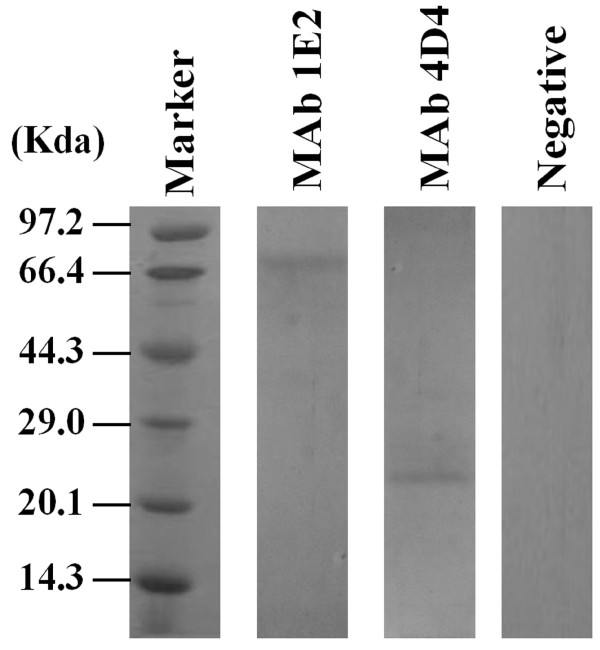
**The MAb against PHE-CoV were identified by Western blot analysis.** Lane 1: Prestained Protein Marke (TaKaRA, code: 530A, Dalian, P. R. China,) Lane 2: The purities of MAb 1E2 were used in Western blot analysis, which recognizes the S Protein of PHE-CoV. Lane 3: The purities of MAb 4D4 were used in Western blot analysis, which recognizes the HE Protein of PHE-CoV. Lane 4: The SP2/0 cells was injected into Balb/C nude mice and the mouse abdominal dropsy, and the purities were used negative control.

The MAb of anti-PHE-CoV were used in Western blot analysis to identify the 4D4 MAb, which recognizes the HE protein, and the 1E2 MAb, which recognizes S protein (Figure 3) and was stored at −80°C until use.

### The sandwich enzyme-linked immunosorbent assay (ELISA)

**Figure 4:**
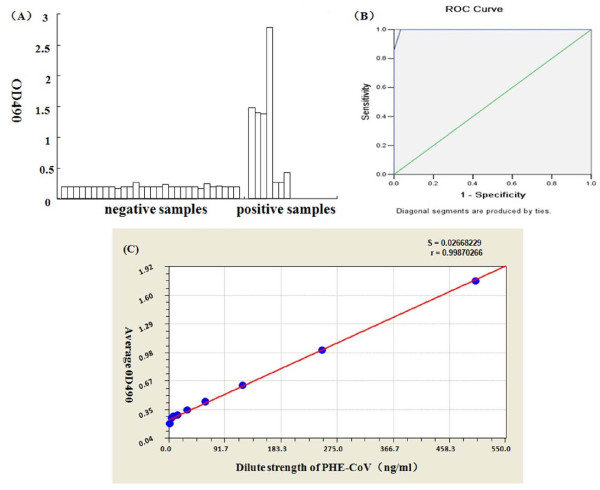
**The virus samples were tested by enzyme-linked immunosorbent assay (ELISA).** Sample numbers 1–30 were negative and 31–37 were positive. **A**, a histogram of ELISA results from both positive and negative virus samples; **B**, SPSS 13.0 for Windows was used to create a ROC curve to evaluate the threshold value; **C**, the standard curve was drawn by CurveExpert software with the PHE-CoV concentration on the X-axis and the average OD490 value on the Y-axis.

Thirty negative and seven positive virus samples were detected by ELISA (Figure 4-A). The threshold value of 0.263 was identified using a ROC curve (Figure 4-B). An OD_490_>0.263 indicated a positive result and ≤0.263 a negative result. Additionally, PHE-CoV was diluted to 500 ng/ml, 250 ng/ml, 120 ng/ml, 60 ng/ml, 30 ng/ml, 15 ng/ml, 7.5 ng/ml, 3.75 ng/ml, and 1.875 ng/ml with PBS, which was detected by ELISA. The standard curve was drawn by CurveExpert software with PHE-CoV concentration on the X-axis and the average OD_490_ value on the Y-axis (Figure 4-C). The linear regression constant R^2^ was 0.9987, and the linear detection range was 3.75-500 ng/ml. Good linearity was observed, as the lowest detection limit was 3.75 ng/ml.

### Colloidal gold and gold-labeled protein identification

**Figure 5:**
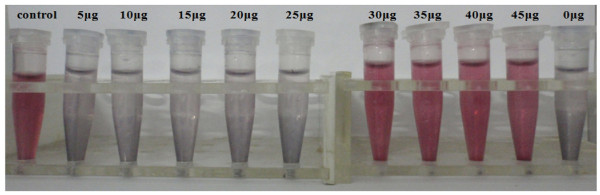
**The optimal antibody concentration.** The colloidal gold solution was made with different concentration of MAb 4D4. The color of the tubes without sufficient protein changed from red to blue, and when the amount of protein added to the tube exceeded the minimum needed, the color remained unchanged.

To determine the optimal concentration of monoclonal antibody with colloidal gold, different concentrations of 4D4 MAb were added in11 tubes with 1 ml colloidal gold solution each. As shown in Figure 5. The color of the tubes without sufficient protein changed from red to blue, whereas the color of the tubes remained unchanged if the amount of protein exceeded the minimum needed. The amount of protein in the lowest colloidal gold concentration tube, which remained red, was increased by 20% when the optimal antibody concentration was reached.

**Figure 6:**
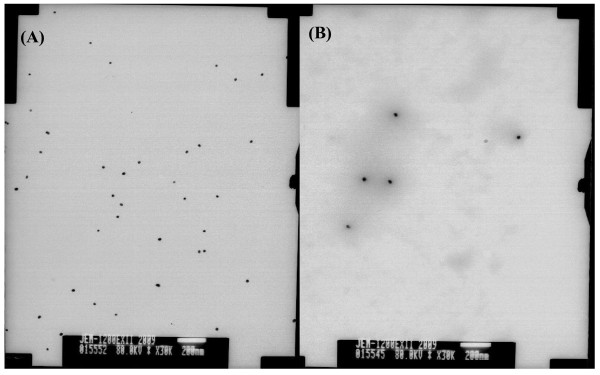
**The colloidal gold and gold-labeled proteins were observed by electron microscopy.** The results of transmission electron microscope imaging of colloidal gold and gold-labeled proteins. **A**: colloidal gold (×30,000), **B**: gold-labeled MAb 4D4 (×30,000).

The appearance of the colloidal gold solution was deep red and translucent, with a bright color. A bright band was visible when the test card faced the sun. The colloidal gold particles were consistent in size and uniformly distributed, with a mean diameter of about 30 nm (Figure 6-A) when observed under a transmission electron microscope. The colloidal gold-labeled MAb 4D4 was observed with the transmission electron microscope to be evenly distributed, and the particle size was consistent. The colloidal gold particles had a visible clear space around the halo, then the surface of proteins and other adsorbed particles (Figure 6-B).

### Specificity and sensitivity of the immunochromatographic strip

**Figure 7:**
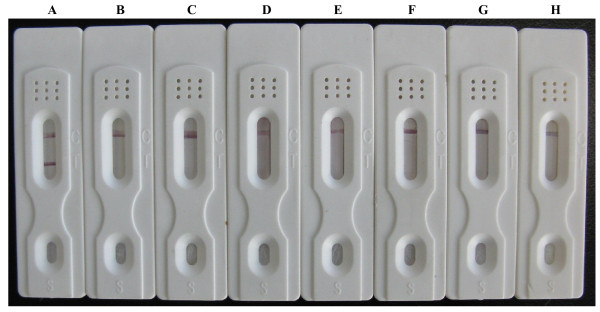
**The specificity of the immunochromatographic strips.** To determine the specificity of the immunochromatographic strip, PHE-CoV(**A**) was simultaneously tested with TGEV(B), PEDV(C), HCV(D), PRV(E), BCV(F), MHV(G) and HCV-OC43(H) using the immunochromatographic strips. All of the non-PHE-CoV samples tested showed one strong band on the control line (**B**, TGEV; **C**, PEDV; **D**, HCV; **E**, PRV; **F**, BCV; **G**, MHV; **H**, HCV-OC43), while A (PHE-CoV-positive virus) displayed an additional band on the test line.

**Figure 8:**
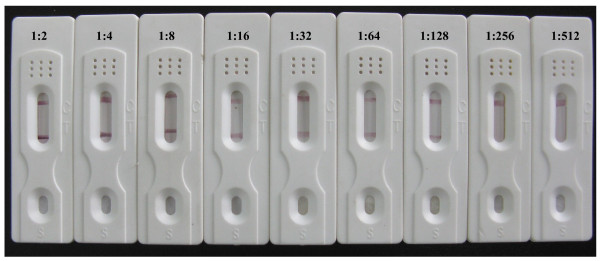
**The sensitivity of the immunochromatographic strip.** To detect the sensitivity of the immunochromatographic strip, PHE-CoV (128 units of HA) was diluted to 1:2, 1:4, 1:8, 1:16, 1:32, 1:64, 1:128, 1:256, and 1:512 with 10 mmol/l borate buffer solution (pH 8.0) were tested.

To determine the specificity of the immunochromatographic strip, PHE-CoV was simultaneously tested with TGEV, HCV, PEDV, PRV, BCV, MHV and HCV-OC43 using the immunochromatographic strips. Clearly, while each of the other samples resulted in one strong band on the control line, PHE-CoV displayed an additional band on the test line of the immunochromatographic strips (Figure 7). PHE-CoV was detected at different dilution strengths and repeated in triplicate with the immunochromatographic strip. The results are shown in Figure 8. When the antigen were diluted from 1:2 to 1:64 (from 128 to 2 HA units, respectively), the reaction on the test and control lines were observed. Thus, the sensitivity of the PHE-CoV colloidal gold immunochromatographic test was determined to be 2 units of HA antigens.

### Reproducibility and stability of the immunochromatographic strip

This procedure was repeated five times to detect the PHE-CoV of HA units at 16 by the same batch and then by five different batches to determine reproducibility. These results (Table 1) showed little variability within the same batch or in different batches, demonstrating that the detection of the virus is highly reproducible. In addition, the stability of the immunochromatographic strip under various storage conditions was determined. The results are shown in Table 2, they were stored at room temperature for 6 months with a reduction in sensitivity of 50% and at 4°C for more than 12 months with no loss of sensitivity or specificity for the detection of PHE-CoV.

**Table 1:** Reproducibility of the immunochromatographic strips (ICS) for the detection of PHE-CoV

**ICS**	**No. of positive results**	**Positive**	**CV**^**a**^
Same batch	50	100%	1.69%
Different batches	48	96%	
	50	100%	
	50	100%	
	50	100%	
	49	98%	

^a^ CV = coefficient of variation. Number of serum samples in each batch was 10, and each batch was tested 5 times.

**Table 2:** The stability of the immunochromatographic strip under various storage conditions

**Storage time**	**Temp (°C)**	**PHE-CoV dilution strength (HA units/ml)**	**Negative samples**
0 mo	4	1:16	—
	RT	1:16	—
2 mo	4	1:16	—
	RT	1:16	—
4 mo	4	1:16	—
	RT	1:16	—
6 mo	4	1:16	—
	RT	1:16	—
8 mo	4	1:16	—
	RT	1:8	—
10 mo	4	1:16	—
	RT	1:4	—
12 mo	4	1:16	—
	RT	1:4	—

### Agreement between the immunochromatographic strip and reference methods

To evaluate the agreement between the immunochromatographic strip and reference methods, the 178 clinical samples from deceased piglets were detected by sandwich ELISA, RT-PCR, and the immunochromatographic strip. The results are shown in Table 3. Of the 178 clinical samples, 88 were positive and 90 were negative using the immunochromatographic strip, while 90 were positive and 88 were negative using ELISA and RT-PCR. Thus, the specificity and sensitivity of the immunochromatographic strip, as compared with ELISA and RT-PCR, were 100% and 97.78%, respectively. There were excellent agreement (Kappa = 0.976) between the immunochromatographic strip to ELISA and RT-PCR.

**Table 3:** Agreement between the immunochromatographic strip (ICS) and reference methods

**ICS**	**ELISA**^**a**^			**RT-PCR**^**b**^			**Total**
	**Positive**	**Negative**	**Kappa**	**Positive**	**Negative**	**Kappa**	
Positive	88	0	0.976	88	0	0.976	88
Negative	2	88		2	88		90
Total	90	88		90	88		178

^a^Relative to ELISA, specificity of ICS: 88/88 × 100% = 100%; sensitivity of ICS: 88/90 × 100% = 97.78%; Po = (88 + 88)/178 = 98.88%;

Pe = [(90/178) × (88/178) + (88/178) × (90/178)] = 0.50; Kappa = (Po-Pe)/(1-Pe) = 0.976.

^b^Relative to RT-PCR, specificity of ICS: 88/88 × 100% = 100%; sensitivity of ICS: 88/90 × 100% = 97.78%; Po = (88 + 88)/178 = 98.88%;

Pe = [(90/178) × (88/178) + (88/178) × (90/178)] = 0.50; Kappa = (Po-Pe)/(1-Pe) = 0.976.

### Clinical application of the immunochromatographic strip

The immunochromatographic strip was next applied to the diagnosis of PHE-CoV infection in the Jilin Province. Characterization of these samples revealed that 207 out of 468 swab samples were positive for PHE-CoV infection (Table 4). Notably, the positive rate ranged from 61.54% in the Jilin district to 17.95% in the Songyuan district. These findings suggest that PHE-CoV is commonly transmitted in the Jilin province, and the immunochromatographic strip can be used for the detection and differentiation of PHE-CoV in clinical diagnosis.

**Table 4:** Diagnosis of PHE-CoV infection in the field using the immunochromatographic strip

**District of Pig Farm**	**No. of nasal cavity or throat swabs**	**No. of positive samples**	**Percent Positive**
Baishan	78	29	37.18
Changchun	78	37	47.44
Jilin	78	48	61.54
Liaoyuan	78	32	41.03
Siping	78	47	60.26
Songyuan	78	14	17.95
Total	468	207	44.23

## Discussion

PHE-CoV belongs to group 2 of the Coronaviridae family, a group characterized by the presence of a gene encoding the HE protein [26]. Nucleotide sequence analysis of the region covering the S2 probe revealed 92.6% nucleotide sequence homology to BCV and 91.9% homology to HCV-OC43 [27]. Although PHE-CoV causes two distinct clinical syndromes in pigs, only one serotype of the virus is known to exist. Outbreaks of PHE-CoV-associated disease are now on the rise in many countries, inflicting considerable economic damage to the pig industry. Remarkably, these recent isolates showed a high degree of genetic and antigenic homology with the 1962 reference strain PHE-CoV-67 N [10]. Currently, immunohistochemistry (IHC) for PHE-CoV, or molecular tools such as RT-PCR, enable the detection of specific CoV RNA sequences from infected tissues [15,28]. However, these detection techniques usually require special primers, a working laboratory, skilled technicians, and specialized equipment, making rapid and on-site detection of viruses in the field difficult. Therefore, the development of a sensitive, specific, and easily performed assay is crucial for the rapid detection and surveillance of PHE-CoV infection and transmission.

Colloidal gold immunochromatographic assay (GICA) is convenient, is rapid, has high specificity and sensitivity, and can be performed either without instruments or with only a simple instrument, making it suitable for clinical diagnosis and drug testing purposes in almost any context [29-31]. Colloidal gold is a negatively charged hydrophobic rubber particle that maintains a stable colloidal system through electrostatic repulsion [22]. The key to successful colloidal gold labeling lies in the preparation of a homogeneous mixture of monodispersed colloidal gold particles [32]. The microwave oven method was chosen in this experiment because it provides even heating without manual shaking, creating colloidal gold particles that are consistent in size and uniformly distributed, with a mean diameter of about 30 nm under transmission electron microscope. In the preparation of gold markers, proteins and gold glue particles should also be present in an appropriate ratio. If too many antibodies are added, the amount of free antibodies instead of gold-labeled antibodies will increase, which will result in a decreased titer of gold-labeled MAb 4D4, affecting the test’s sensitivity. Conversely, if too few antibodies are added, it is easy for the colloidal particles to aggregate and precipitate, especially during purification by ultracentrifugation. Therefore, it is essential to add sufficient colloidal gold antibodies to maintain stability.

The specificity and sensitivity of the immunochromatographic strip are largely dependent on the following factors. First, the quality of the MAbs used in the strip test is crucial for the specificity and sensitivity of the strip. In this experiment, both of the MAbs used recognize the HE and S proteins of PHE-CoV and have a high affinity for their respective antigen epitopes. Analysis of the specificity showed the strip to be specific for the detection of PHE-CoV, because it reacted with neither two coronaviruses (TGEV and PEDV) in pigs nor with other group 3 viruses of the Coronaviridae family (BCV, HCV-OC43 and MHV), the common virus HCV, or PRV, to which the clinical symptoms of PHE-CoV are similar. In addition, careful selection of a membrane is critical for high specificity, sensitivity, and rapid detection as the wicking rate and speed of liquid diffusion on the membrane (Millipore Corp SHF135, a liner). The membrane used in this experiment was nitrocellulose membrane (Millipore Corp SHF135, a liner) with a membrane pore size of 0.5-1.0 μm, IgG binding force constant of 60–100 μg/cm2, and a chromatography rate of 15–20 s/cm. The membrane ensures antibody absorption and the leaching and binding of gold-labeled proteins. These advantages enable the test results to be sensitive and easy to read [33].

PHE-CoV is able to replicate in the upper respiratory tract with or without producing clinical signs. PHE-CoV can be isolated from the nasal cavity, trachea, brain, and lungs of diseased or healthy pigs [34-36] and the brain had the highest detection rate by nested PCR and RT-PCR [15]. Because of RT-PCR and ELISA are also widely applied in clinical practice due to its high sensitivity and specificity [37,38]. Thus, a lot of brain tissue samples were collected from deceased piglets with suspected PHE-CoV infection, and using RT-PCR and ELISA as reference test, the relative specificity and sensitivity of the immunochromatographic strip were determined to be 100% and 97.78%, respectively. There was an excellent agreement among the immunochromatographic strip to ELISA and RT-PCR (Kappa = 0.976). The quantifying test result indicated the sensitivity of the strip test to be close to, or slightly less than, that of ELISA and RT-PCR. However, the ELISA and RT-PCR assay each step of the procedure can strongly influence the result if not performed well. The procedure must be strictly followed, with good quality control inside and outside the laboratory, and reagents that ensure the test quality must be selected. Although ELISA and RT-PCR was developed for the detection of PHE-CoV with high specific and sensitivity, it is not suitable for use outside of the research laboratory. In contrast, the procedure for the immunochromatographic strip described in this paper is less laborious and time-consuming than the ELISA and RT-PCR method. Taken together, the results suggested that an immunochromatographic strip was developed with high sensitivity and specificity for detecting PHE-CoV, which could be used for clinical applications.

PHE-CoV is excreted for 8–10 days in oronasal secreions [34,39], with transmission occurring through exposure to nasal secretions. Thus, a total of 468 nasal cavity or throat swabs were collected from approximately 1- to 3-week-old piglets, with vomiting and neurological symptoms consistent with PHE-CoV infection, and application of the immunochromatographic strips for diagnosis of PHE-CoV infection in the Jilin province. The positive rate ranged from 61.54% in the Jilin district to 17.95% in the Songyuan district. Notably, the PHE-CoV positive rate was 41.03% in Liaoyuan district in the current study. However, the serum antibody positive rate was only 6.5% in the previous study [40]. This may be associated with the samples collected from different herds. These findings suggest that PHE-CoV is commonly transmitted in these districts in the Jilin province. Notably, specific pathogen-free (SPF) pigs, derived from germ-free pigs and given artificial milk, have been introduced extensively in many farms in this area [15,41]. Because these animals lack antibodies to certain pathogenic agents, including PHE-CoV, their susceptibility to these agents may be greater. Therefore, reliable testing methods are important for the diagnosis and prevention of PHE-CoV disease, which can cause fatal outbreaks in PHE-CoV seronegative farms.

## Conclusion

From the results of clinical signs, an immunochromatographic strip was developed for detecting PHE-CoV. The immunochromatographic strip is a simple and quick test that requires no special training to use, and the sensitivity and specificity of the strip were similar to the ELISA and RT-PCR test. This test would be a valuable addition to clinical detection techniques for PHE-CoV, especially in areas where laboratory facilities are not available.

## Abbreviations

PHE-CoV: Porcine hemagglutinating encephalomyelitis coronavirus; HA/HI: Hemagglutination/hemagglutination inhibition; IHC: Immunohistochemistry; nested PCR: Nested-polymerase chain reaction; RT-PCR: Reverse transcriptase-polymerase chain reaction; MAb: Monoclonal antibody; GICA: Colloidal gold immunochromatography; HE: Hemagglutinin-esterase protein (HE); S: Spike glycoprotein; ELISA: Enzyme-linked immunosorbent assay; BSA: Bovine serum albumin; PBS: Phosphate Buffered Saline; NC: Nitrocellulose; TGEV: Transmissible gastro-enteritis virus; PEDV: Pig Epidemic Diarrhea Virus; PRV: Pseudorabies virus; HCV: Hog Cholera Virus; BCV: Bovine coronavirus; MHV: Mouse hepatitis virus; HCV-OC43: Human respiratory coronavirus OC43.

## Competing interests

The authors declare that they have no competing interests.

## Authors’ contribution

KC and KZ carried out most of the experiments and wrote the manuscript. DS and WH participated in the planning of the project. CZ and GW collected the samples. FG and CW conceived of the study and participated in its design and coordination. All authors read and approved the final manuscript.
